# Transcriptional regulators and regulatory pathways involved in prostate gland adaptation to a hypoandrogen environment

**DOI:** 10.1590/1678-4685-GMB-2018-0362

**Published:** 2020-02-14

**Authors:** Umar Nishan, Rafaela da Rosa-Ribeiro, Danilo Marchete Damas-Souza, Guilherme Oliveira Barbosa, Hernandes F. Carvalho

**Affiliations:** ^1^ Universidade de Campinas (UNICAMP) Universidade de Campinas (UNICAMP) Departamento de Biologia Estrutural e Funcional CampinasSP Brazil Departamento de Biologia Estrutural e Funcional, Instituto de Biologia, Universidade de Campinas (UNICAMP), Campinas, SP, Brazil

**Keywords:** Androgens, Arnt, castration, prostate, transcription factors

## Abstract

Anti-androgen therapies, including orchiectomy, are effective at promoting prostate cancer remission, but are followed by progression to the more aggressive castration-resistant prostate cancer (CRPC). Castration promotes gland and tumor shrinkage. However, prostate adaptation to androgen deprivation involves striking parallel events, all requiring changes in gene expression. We hypothesized that transcription factors (TF) and other transcription-related genes are needed to orchestrate those changes. In this work, downstream analysis using bioinformatic tools and published microarray data allowed us to identify sixty transcriptional regulators (including 10 TF) and to integrate their function in physiologically relevant networks. Functional associations revealed a connection between *Arnt*, *Bhlhe41* and *Dbp* circadian rhythm genes with the *Ar* circuitry and a small gene network centered in Pex14, which might indicate a previously unanticipated metabolic shift. We have also identified human homologs and mapped the corresponding genes to human chromosome regions commonly affected in prostate cancer, with particular attention to the *PTEN*/*HHEX*/*MXI1* cluster at 10q23-25 (frequently deleted in PCa) and to MAPK1 at 22q11.21 (delete in intermediate risk but not in high risk PCa). Twenty genes were found mutated or with copy number alterations in at least five percent of three cancer cohorts and six of them (PHOX2A, NFYC, EST2, EIF2S1, SSRP1 and PARP1) associated with impacted patient survival. These changes are specific to the adaptation to the hypoandrogen environment and seem important for the progression to CRPC when mutated.

## Introduction

Prostate diseases in general and prostate cancer (PC) in particular are major concerns of public health care. One eighth to one sixth of males will develop PC and experience the risk of prostate cancer progression if not properly diagnosed, monitored and treated. Molecular markers for diagnosis and disease progression risk assessment are extremely necessary. One of the palliative treatments for advanced prostate cancer is androgen blockade, achieved by chemical or surgical castration. Besides the psychological and physiological side effects, the risk of androgen blockade resides in the common progression to the highly malignant and life-threatening form of castration-resistant prostate cancer (CRPC). Several mutations and chromosomal rearrangements have been associated with PC and CRPC. Recently, a series of chromosomal translocations including frequent bridging and rearrangements was described and demonstrated to occur in a few steps and to affect a series of important tumor suppressors (Baca *et al.*, 2013). Nonetheless, the linear progression among progressive stages has been questioned, as metastatic lesions are clonally derived, show signatures particular to each affected individual and might result from less advanced local primary lesions (Holcomb *et al.*, 2009, Haffner *et al.*, 2013).

Androgens, acting through the androgen receptor (AR), are required for prostate development and normal function (Roy *et al.*, 1998). Hence, surgical or chemical castration defines a hypoandrogenic state in which a series of events sum up to promote organ (and tumor) shrinkage; androgen deprivation/blocking is the first line therapy for advanced prostate cancer. Gain-of-function mutations enabling the AR to recover activity in the hypoandrogen environment have been associated with the progression to CRPC (Feldman and Feldman, 2001), and include mutations, deletions and inversions at the ligand binding site leading to ligand-independent activation (Nyquist *et al.*, 2013), and gene amplifications. In spite of these AR-centered modifications, a series of genes has been implicated in prostate cancer progression, including overall changes in gene expression (Abate-Shen and Shen, 2000, Tomlins *et al.*, 2007, Shen and Abate-Shen, 2010) and complex chromosomal rearrangements frequently involving *PTEN*, *NKX3.1*, *SPOP, CHD1, TP53, MAP3K7, FOXP1* and the *T2-ERG* fusion (Baca *et al.*, 2013).

Furthermore, we consider that other physiological aspects of the adaptation to the hypoandrogen environment might be corrupted in cancer cells, and cooperate in establishing the selective pressure that contributes to the clonality of cells harboring chromosomal changes in general and AR modifications in particular.

Although epithelial cell apoptosis is a major event in prostate regression occurring in response to castration, it is not the sole event. For instance, remarkable reprogramming of immune system cells (Desai *et al.*, 2004) and smooth muscle cells (Antonioli *et al.*, 2004, 2007) as well as reorganization of the extracellular matrix (Vilamaior *et al.*, 2000) have been described and associated with a re-defined functional state and immune barrier system. Additionally, we have reported the occurrence of desquamation as an additional phenomenon contributing to epithelial cell deletion (Rosa-Ribeiro *et al.*, 2014a), and a relevant role for two macrophage subpopulations in both (a) the induction of epithelial cell death (Barbosa *et al.*, 2019) and (b) the clearance of cell corpses and maintenance of the non-inflammatory status (Silva *et al.*, 2018).

However, little has been studied beyond the induction of apoptosis in epithelial cells. Progress has been made in terms of showing that epithelial expression of the AR is not necessary for epithelial cell death (Kurita *et al.*, 2001), while the remaining cells develop resistance to androgen deprivation and preserve a differentiation-immature signature (Rosa-Ribeiro *et al.*, 2014b).

The rat ventral prostate has been a valuable and robust *in vivo* model system to explore androgen regulation of gene expression (Wang *et al.*, 1997, Kwong *et al.*, 1999, Desai *et al.*, 2004). The ventral prostate responds to androgen withdrawal with increased epithelial cell apoptosis, whereas the dorsolateral lobes show negligible cell death (Kwong *et al.*, 1999). The responsiveness of the ventral prostate to androgens is also characterized by the number of differentially expressed genes after castration as compared to the dorsolateral lobe (1496 vs. 256 genes, respectively) (Desai *et al.*, 2004). Most of these changes take place in the hypoandrogen environment, and are triggered by disengaging AR signaling.

We hypothesized that other transcription factors (TF) and transcriptional regulators (TR) are co-opted to coordinate the sequential changes observed in the gland after castration. In this work, we explore this idea in an attempt to find new genes, unforeseen metabolic process and chromosomal hotspots that might reveal possible connections between gland physiology under androgen deprivation promoted by castration and progression to CRPC after hormone therapies.

We have used bioinformatics to (a) select class-specific genes from a published list of genes and ESTs differentially expressed in response to castration and androgen supplementation after DNA microarray analysis, (b) to identify the regulatory networks in which the selected genes are involved, (c) to map the homologs of rat genes to human chromosomes, and (d) to find mutations and/or copy number alterations and changes in patient survival.

Accordingly, this study has unveiled a list of TF and TR genes and a series of unexplored physiological pathways, such as circadian rhythms (genes Arnt/Bhlhe41/Dpb) and peroxisome biogenesis (Pex14), hitherto neglected pathways in prostate biology. We also correlated the selected genes with chromosomal regions commonly deleted in prostate cancer, such as 10q23, which contains the *PTEN*/*HHEX*/*MXI1* gene cluster, and 22q11.21, harboring the MAPK1 gene, found 20 genes mutated in at least 5% of three patient cohorts and six genes affecting patient survival when mutated.

## Materials and Methods

The microarray data from Desai *et al.* (2004) reported 1496 genes/ESTs differentially expressed in response to castration and testosterone supplementation. The list of gene bank accession IDs for all genes and ESTs was loaded into DAVID v6.7. Gene IDs and biological annotations are highly redundant within the vast array of public databases. The DAVID knowledge base collects and integrates various gene identifiers as well as more than 40 well-known publicly annotation categories, which are then centralized by the internal DAVID identifier in a non-redundant manner. A significant portion of input gene IDs failed to be mapped and were then processed using the gene ID conversion tool. All the identified IDs/gene names were listed by the gene name batch viewer. We further processed the identified IDs for the identification of functional annotations centered on TFs and TRs, and the identified genes were further studied to find their functional annotation clustering and possible integration in known biological functions.

The TFs/TRs were also studied for possible functional associations using the Ingenuity Pathway Analysis (IPA) software, with filtering for information in the rat, and choosing only direct interactions.

The human homologs to the rat genes were searched manually using the NCBI database, and their chromosomal location was used to map them to the human ideogram.

Finally, we assessed the cBioPortal (cbioportal.org) and checked three cohorts of prostate adenocarcinomas for the existence of mutations and/or copy number alterations and possible effect on patient survival (Armenia *et al.*, 2018, Liu *et al.*, 2018, Abida *et al.*, 2019).

A limit of 5% mutations and a log rank test p-value smaller than 0.05% were set for each analysis, respectively.

## Results

### Data processing

Using the gene accession conversion tool of DAVID v6.7, the program managed to convert 468 IDs from the list of 1477 total unique user IDs. The number of genes identified was similar to that obtained in the original work (Desai *et al.*, 2004). Out of the 468 IDs, DAVID identified 60 TFs/TRs. Table 1 lists the detailed annotation and functional enrichment information that was retrieved using the terms TFs/TRs. The chromosomal location for each gene was determined using the NCBI databank. Twenty-two genes were identified as transcription factors (bold in Table 1).

**Table 1:** Genes with accession numbers, names and functions and chromosomal location in the rat chromosomes as well as the chromosomal location of the human homologs. Known and putative transcription factors are bold-faced.

S.No	Gene Accession Number	Gene Name	Abbreviation	Rat Chromosome location	Location of the human homolog	Gene Function
1	NM_017259	B-cell translocation gene 2, anti-proliferative	Btg2	13q13-q31	1q32	Regulation of transcription
2	NM_057109	BarH-like homeobox 1	**Barhl1**	3p12	9q34	Regulation of transcription, transcription factor activity
3	NM_013154	CCAAT/enhancer binding protein (C/EBP), delta	**Cebpd**	11	8p11.2-p11.1	Regulation of transcription from RNA polymerase II promoter, transcription factor activity
4	NM_012543	D site of albumin promoter (albumin D-box) binding protein	**Dbp**	1q22	19q13.3	Transcription factor activity, Basic-leucine zipper (bZIP) transcription factor
5	NM_020083	GTPase activating Rap/RanGAP domain-like 1	Ralgapa1	6q23	14q13.2	Regulation of transcription
6	NM_012855	Janus kinase 3	Jak3	16p14	19p13.1	Regulation of transcription, transcription factor binding
7	NM_013160	MAX interactor 1	Mxi1	1q55	10	DNA binding, transcription repressor activity, transcription regulator activity
8	NM_022856	Ngfi-A binding protein 1	Nab1	9q22	2q32.3-q33	Transcription repressor activity, transcription regulator activity
9	NM_019275	SMAD family member 4	**Smad4**	18q12.3	18q21.1	Transcription factor complex, transcription activator activity, transcription regulator activity
10	NM_017359	RAB10, member RAS oncogene family	Rab10	6q12	2p23.3	Regulation of transcription, transcription factor binding
11	NM_021693	SNF1-like kinase	Sik1	20p12	21q22.3	Transcription repressor activity, transcription regulator activity
12	NM_012903	Acidic (leucine-rich) nuclear phosphoprotein 32 family, member A	Anp32a	8q24	15q23	Regulation of transcription
13	NM_031018	Activating transcription factor 2	**Atf2**	3q23	2q32	Transcription factor activity, transcription activator, Basic-leucine zipper (bZIP) transcription factor, Cyclic AMP-dependent transcription factor ATF-2, bZIP transcription factor
14	M64780	Agrin	Agrn	5q36	1p36.33	Regulation of transcription, regulation of transcription from RNA polymerase II promoter
15	NM_012907	Apolipoprotein B mRNA editing enzyme, catalytic polypeptide 1	Apobec1	4q42	12p13.1	Posttranscriptional regulation of gene expression
16	AF015953	Aryl hydrocarbon receptor nuclear translocator-like	**Arntl**	1q34	11p15	Positive regulation of transcription, transcription factor activity
17	AF009329	Basic helix-loop-helix family, member e41	**Bhlhe41**	4q43	12p12.1	Transcription regulator activity
18	NM_017338	Calcitonin/calcitonin-related polypeptide, alpha	Calca	1q34	11p15.2	Regulation of transcription
19	X83579	Cyclin-dependent kinase 7	Cdk7	2q12	5q12.1	Transcription factor complex, transcription regulation
20	NM_012698	Dystrophin, muscular dystrophy	Dmd	Xq22	Xp21.2	Regulation of transcription
21	NM_012754	Estrogen receptor 2 (ER beta)	**Esr2**	6q24	14q23.2	Transcription factor activity
22	NM_031041	General transcription factor IIB	**Gtf2b**	2q44	1p22-p21	transcription initiation, transcription factor complex, Transcription factor TFIIB related
23	J05181	Glutamate-cysteine ligase, catalytic subunit	Gclc	8q31	6p12	Regulation of transcription
24	NM_021592	Heart and neural crest derivatives expressed 1	**Hand1**	10q22	5q33	DNA binding, transcription factor activity, transcription cofactor activity, transcription coactivator activity, transcription factor binding, enzyme binding, transcription regulator activity, bHLH transcription factor binding
25	NM_024385	Hematopoietically expressed homeobox	**Hhex**	1q53	10q23.33	DNA binding, transcription factor activity, eukaryotic initiation factor 4E binding, general transcriptional repressor activity, transcription regulator activity, translation initiation factor binding, sequence-specific DNA binding,
26	NM_032070	High mobility group AT-hook 2	Hmga2	7q22	12q15	Regulation of transcription
27	NM_031787	Homeodomain interacting protein kinase 3	Hipk3	3q32	11p13	Regulation of transcription
28	NM_019356	Eukaryotic Translation initiation factor 2, subunit 1 alpha	Eif2s1	6q24	14q23.3	Posttranscriptional regulation of gene expression
29	NM_013060	Inhibitor of DNA binding 2	**Id2**	6q16	2p25	Regulation of transcription factor activity,
30	NM_053355	Inhibitor of kappa light polypeptide gene enhancer in B-cells, kinase beta	Ikbkb	16q12.5	8p11.2	Regulation of transcription factor activity, positive regulation of NF-kappaB transcription factor activity
31	NM_012591	Interferon regulatory factor 1	Irf1	10q22	5q31.1	DNA binding, transcription factor activity, sequence-specific DNA binding
32	NM_053842	Mitogen activated protein kinase 1	Mapk1	11q23	22q11.21	Transcription factor binding, positive regulation of transcription
33	NM_017322	Mitogen-activated protein kinase 9	Mapk9	10q22	5q35	Regulation of transcription
34	NM_053718	myeloid/lymphoid or mixed-lineage leukemia (trithorax homolog, Drosophila); translocated to, 3	Mllt3	5q32	9p22	Regulation of transcription
35	U68726	Neogenin homolog 1 (chicken)	Neo1	8q24	15q22.3-q23	Transcription regulator activity
36	NM_012866	Nuclear transcription factor-Y gamma	**Nfyc**	5q36	1p32	DNA binding, transcription factor activity, sequence-specific DNA binding,
37	NM_053869	Paired-like homeobox 2a	**Phox2a**	1q32	11q13.2	DNA binding, transcription factor activity, sequence-specific DNA binding
38	AB017544	Peroxisomal biogenesis factor 14	Pex14	5q36	1p36.22	Transcription cofactor activity, transcription corepressor activity, transcription factor binding, transcription repressor activity,
39	NM_031606	Phosphatase and tensin homolog	Pten	1q41-q43	10q23.3	Posttranscriptional regulation of gene expression,
40	NM_013063	poly (ADP-ribose) polymerase 1	Parp1	13q26	1q41-q42	Transcription regulation
41	NM_053949	potassium voltage-gated channel, subfamily H (eag-related), member 2	Kcnh2	4q11	7q36.1	Transcription regulation
42	NM_019243	prostaglandin F2 receptor negative regulator	Ptgfrn	2q34	1p13.1	Posttranscriptional regulation of gene expression
43	NM_031149	proteasome (prosome, macropain) 26S subunit, ATPase, 5	Psmc5	10q32.1	17q23.3	Regulation of transcription, transcription factor binding
44	NM_031528	Retinoic acid receptor, alpha	**Rara**	10q31	17q21	Transcription factor activity, transcription cofactor activity, transcription coactivator activity, Transcription, transcription
45	AJ223083	Retinoid X receptor gamma	**Rxrg**	13q24	1q22-q23	Transcription factor activity, transcription regulator activity
46	L29259	Similar to transcription elongation factor B (SIII), polypeptide 1; transcription elongation factor B (SIII), polypeptide 1	Tceb1	5q11	8q21.11	RNA polymerase II transcription factor activity, transcription elongation regulator activity, regulation of transcription
47	NM_030835	Stress-associated endoplasmic reticulum protein 1	Serp1	2q31	3q25.1	Posttranscriptional regulation of gene expression,
48	L08814	Structure specific recognition protein 1	Ssrp1	3q24	11q12	DNA binding, transcription regulator activity
49	NM_053800	Thioredoxin 1	Txn1	5q24	9q31	Regulation of transcription
50	U30789	Thioredoxin interacting protein	Txnip	2q34	1q21.1	Regulation of transcription
51	NM_012887	Thymopoietin	Tmpo	7q13	12q22	Regulation of transcription
52	J03819	Thyroid hormone receptor beta	**Thrb**	15p16	3p24.2	DNA binding, double-stranded DNA binding, transcription factor activity
53	U54632	Transmembrane protein 215; similar to Ubiquitin-conjugating enzyme E2 I (Ubiquitin-protein ligase I) (Ubiquitin carrier protein I) (SUMO-1-protein ligase)	Ube2i	10q12	16p13.3	transcription factor binding, specific transcriptional repressor activity, transcription regulator activity, bHLH transcription factor binding
54	NM_013091	Tumor necrosis factor receptor superfamily, member 1a	Tnfrsf1a	4q42	12p13.2	Regulation of transcription
55	NM_053928	Ubiquitin-conjugating enzyme E2N; similar to ubiquitin-conjugating enzyme E2N (homologous to yeast UBC13)	Ube2n	7q13	Xq27	Regulation of transcription factor activity, regulation of transcription
56	NM_012555	v-ets erythroblastosis virus E26 oncogene homolog 1 (avian)	**Ets1**	8q21	11q23.3	Transcription factor activity, transcription regulator activity, sequence-specific DNA binding
57	NM_017058	Vitamin D (1, 25- dihydroxyvitamin D3) receptor	**Vdr**	7q36	12q13.11	DNA binding, transcription factor activity, transcription factor binding
58	X52590	Zinc finger protein 36, C3H type-like 1	**Zfp36l1**	6q24	14q22-q24	Posttranscriptional regulation of gene expression
59	AF072439	Zinc finger protein 37	**Zfp37**	5q24	9q32	Regulation of transcription
60	AF052042	Zinc finger protein 394	**Zfp394**	12p11	7q22.1	Transcription factor activity, Regulation of transcription

### Roles of transcription factors and functional associations among the selected genes

The selected TFs and TRs function in 17 important cellular pathways identified by DAVID (Table 2). Some of the ontogenies were very general (such as “Pathways in cancer” or “Prostate cancer” or “Type II diabetes mellitus”); all but 3 (14/17) included *Mapk1*, and about half (9/17) contained *Mapk9*, related to areas of strong research or hubs in central signaling pathways. The ontogenies also pointed to TGF-β, Toll-like receptors and T-cell receptor signaling pathways. Novel ontogenies implicated the genes *Arnt* and *Bhlhe41* in “Circadian rhythms” and *Ets-1* (E26 oncogene homolog 1) in “Dorso-ventral axis formation”.

**Table 2:** Regulatory pathways involving the selected genes identified by DAVID.

S.No	Pathway	Gene name	Genes count	%	P-Value	Benjamini’s false discovery rate
1	Pathways in cancer	SMAD family member 4 B-cells, kinase beta Mitogen activated protein kinase 1 Mitogen-activated protein kinase 9 Phosphatase and tensin homolog Retinoic acid receptor, alpha Retinoid X receptor gamma transcription elongation factor B (SIII)	8	13.3	4.5E-4	3.3E-2
2	Adipocytokine signaling pathway	B-cells, kinase beta Mitogen-activated protein kinase 9 Retinoid X receptor gamma Superfamily, member 1a	4	6.7	3.5E-3	1.2E-1
3	Pancreatic cancer	SMAD family member 4 B-cells, kinase beta Mitogen activated protein kinase 1 Mitogen-activated protein kinase 9	4	6.7	3.8E-3	8.9E-2
4	Type II diabetes mellitus	B-cells, kinase beta Mitogen activated protein kinase 1 Mitogen-activated protein kinase 9	3	5.0	2.1E-2	3.3E-1
5	Acute myeloid leukemia	B-cells, kinase beta Mitogen activated protein kinase 1 Retinoic acid receptor, alpha	3	5.0	2.7E-2	3.3E-1
6	MAPK signaling pathway	Activating transcription factor 2 B-cells, kinase beta Mitogen activated protein kinase 1 Mitogen-activated protein kinase 9 Superfamily, member 1a	5	8.3	3.3E-2	3.3E-1
7	NOD-like receptor signaling pathway	B-cells, kinase beta Mitogen activated protein kinase 1 Mitogen-activated protein kinase 9	3	5.0	3.3E-2	3.0E-1
8	Renal cell carcinoma	Mitogen activated protein kinase 1 Transcription elongation factor B (SIII) E26 oncogene homolog 1 (avian)	3	5.0	4.0E-2	3.1E-1
9	Chronic myeloid leukemia	SMAD family member 4 B-cells, kinase beta Mitogen activated protein kinase 1	3	5.0	4.7E-2	3.2E-1
10	Colorectal cancer	SMAD family member 4 Mitogen activated protein kinase 1 Mitogen-activated protein kinase 9	3	5.0	5.4E-2	3.3E-1
11	Small cell lung cancer	B-cells, kinase beta Phosphatase and tensin homolog Retinoid X receptor gamma	3	5.0	5.6E-2	3.2E-1
12	Circadian rhythm	Aryl hydrocarbon receptor Basic helix-loop-helix family, member e41	2	3.3	5.9E-2	3.1E-1
13	TGF-beta signaling pathway	SMAD family member 4 Inhibitor of DNA binding 2 Mitogen activated protein kinase 1	3	5.0	6.0E-2	2.9E-1
14	Prostate cancer	B-cells, kinase beta Mitogen activated protein kinase 1 Phosphatase and tensin homolog	3	5.0	6.5E-2	3.0E-1
15	Toll-like receptor signaling pathway	B-cells, kinase beta Mitogen activated protein kinase 1 Mitogen-activated protein kinase 9	3	5.0	6.5E-2	3.0E-1
16	T cell receptor signaling pathway	B-cells, kinase beta Mitogen activated protein kinase 1 Mitogen-activated protein kinase 9	3	5.0	9.0E-2	3.7E-1
17	Dorso-ventral axis formation	Mitogen activated protein kinase 1 E26 Oncogene homolog 1 (avian)	2	3.3	9.8E-2	3.7E-1

**Figure 1:**
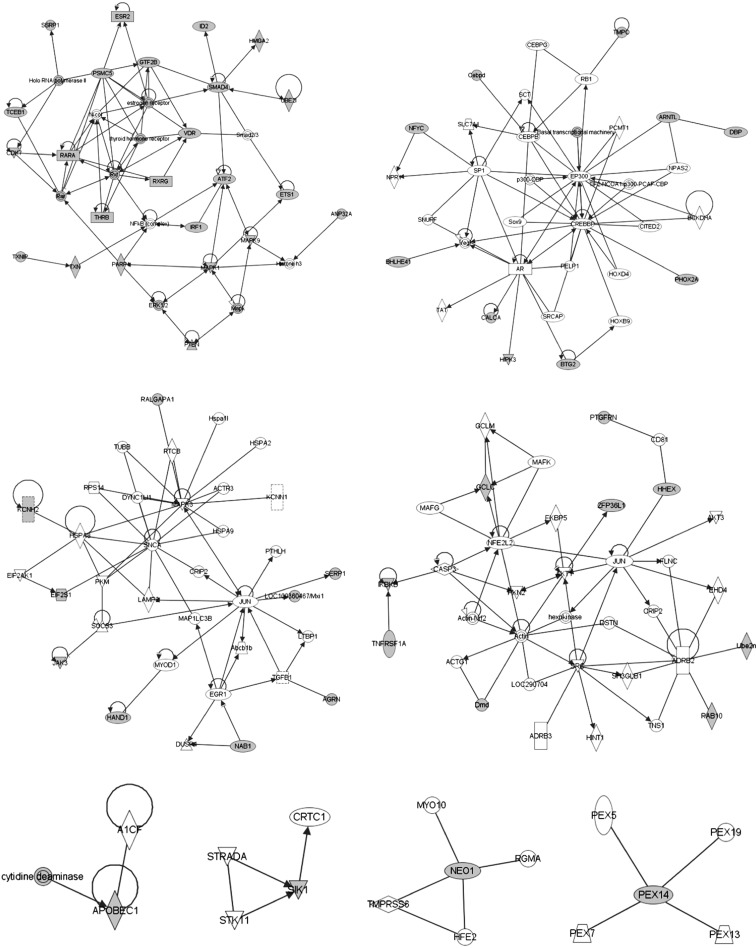
Functional associations among the 60 TF/TR in eight networks, according to IPA. Functional descriptors are presented in Table S1. The genes shown in gray are those identified in this work.

Upon further inspection, using IPA to set the interactions among the 60 genes, we found eight networks corresponding to known pathways (Figure 1; Table S1). They vary in terms of the number of individual nodes, but reveal interesting aspects of the yet-to-be proven physiology of the prostate gland in the hypoandrogen environment. Perhaps not surprisingly, they are ascribed to gene expression regulation, cell death and survival, and also to nucleic acid and carbohydrate metabolism and cancer. They also implicate particular pathways such as estrogen receptor, retinoic acid receptor, thyroid hormone receptor, NFκB signaling, TGF-β and establishing connections with the newly identified genes. It is interesting to note that *Arntl* and *Bhlhe41*, both involved in circadian rhythms (Pathway 12, in Table 2), appeared together in network number 2 (Figure 1). *Arntl* connected to the AR via either p300/EP300 or CREBBP acetyl transferases, and directly do *Dbp*, another circadian rhythm gene. Additionally, IPA retrieved one particularly interesting pathway, peroxisomal biogenesis and function, referenced by network number 8, which is centered on the gene *Pex14*.

### Chromosomal mapping

**Figure 2:**
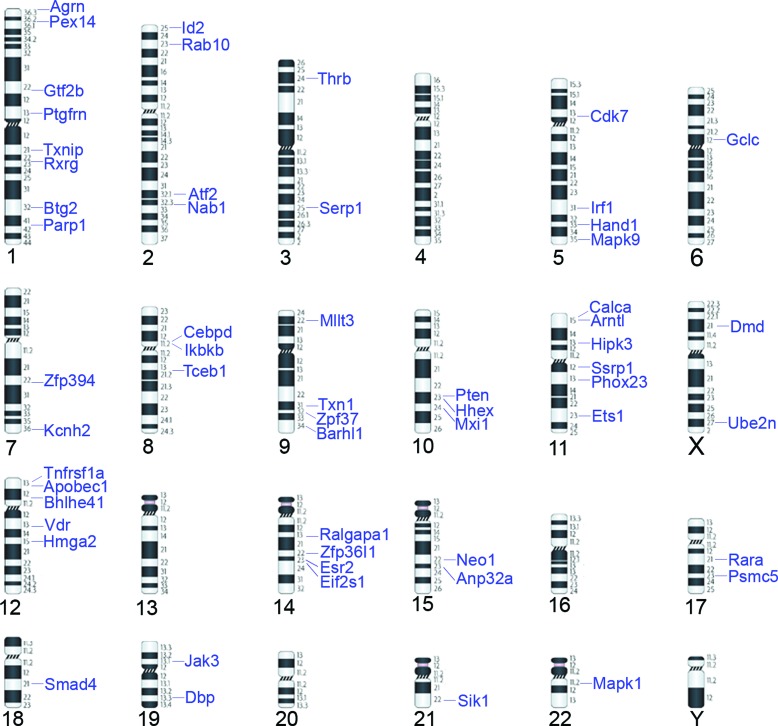
Mapping of the human orthologs of 60 TF/TR rat genes to the human ideogram. Indicated are the regions of copy number gain (blue) and losses (red) reported for healthy human individuals, according to Iafrate *et al*. (2004).

**Figure 3:**
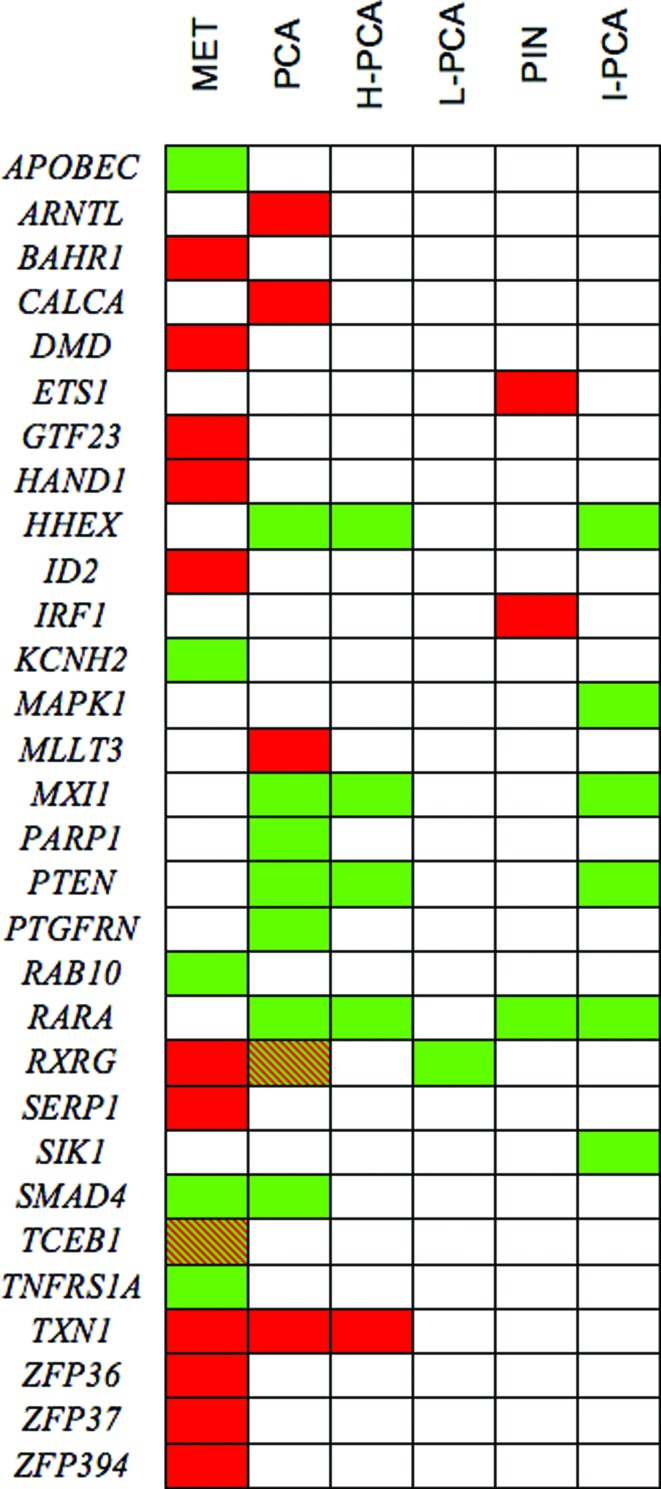
Association of frequently amplified (red), deleted (green) or both (hatched) chromosomal regions harboring 30 of the selected TF/TR in metastatic prostate cancer (MET), prostate cancer (PCA), high grade PCA (H-PCA), low grade PCA (L-PCA), prostate intraepithelial neoplasia (PIN), and intermediate risk PCA (I-PCA). Based on Kim *et al*. (2007) and Ishkamian *et al*. (2009).

We identified the human homolog for each gene (Table 1) and determined their location in the human chromosomes ideogram, also using the NCBI databank. This data set was used to localize each gene in the human ideogram (Figure 2). Chromosome 1 contained eight genes (*AGRN*, *PEX14*, *NFYC*, *GTF2B*, *PTGFRN*, *TXNIP*, *RXRG*, *BTG2*, *PARP1*) and small clusters were observed in 2q (*SERP1*, *ATF2*, *NAB1*), 5q (*IRF1*, *HAND1*, *MAPK9*), 9q (*TXN1*, *ZPF37*, *BARHL1*), 10q (*PTEN*, *HHEX*, *MXI1*), 12p (*TNFRSF1A*, *APOBEC1*, *BHLHE41*) and 14q (*ZFP36L1*, *ESR2*, *EIF2S1*). The 10q23 region included *Pten*, *Hhex* and *Mxi1*. In contrast, not a single gene among the selected 60 mapped to chromosomes 4, 13, 16, 20 and Y. Areas of frequent variation (i.e. gains or deletions) (Iafrate *et al.*, 2004) were included for the determination of proximity to the set of the human homologs of the selected genes. The location of the regions frequently affected by gains/losses in healthy individuals (Iafrate *et al.*, 2004) revealed almost no association with the selected genes (Figure 2). On the other hand, half of the selected genes were mapped to chromosomal regions found to be amplified or deleted in prostatic diseases (Figure 3). Associations were found with metastatic cancer (12/6; gains/losses), localized high (1/4; gain/losses) and low risk (1 loss; *RXRG*), prostate intraepithelial neoplasia (PIN) (2/1; gains/loss) (Kim *et al.*, 2007), and with the intermediate risk prostate cancer (6 losses) (Ishkanian *et al.*, 2009). Remarkably, ETS1 and IRF1 appeared in regions of gains in PIN but not in other disease states, and MAPK1 and SIK1 were located in (or nearby) regions deleted in intermediate-risk cancer.

### Mutation rates and effect on patient survival

PTEN is a tumor suppressor frequently associated with prostate cancer (Abate-Shen and Shen, 2000, Tomlins *et al.*, 2007, Shen and Abate-Shen, 2010, Baca *et al.*, 2013). We found PTEN mutated in 16%, 21% and 33% of the patients for the three cohorts studied. Beside PTEN, only IKBKB was found mutated in at least 5% of the patients in the three cohorts. CEBPD and DMD showed mutations in more than 10% of the patients in one cohort. BTG2, SERP1, CKNH2, RXRG, TXN1P, UBE2I, ZFP37, BARHL1, RALGAPA1, CDK7, PARP1, PTEFGRN, TXN1, TMPO, TNFRSF1A and AGRN were mutated in at least 5% of the patients in at least one cohort. However, more than 50% of the studied genes showed deletions in the three cohorts.

Survival curves existed for two of the three cohorts studied. PHOX2a and NFYC were associated with significant impact on patient survival (P < 0.05) in the first cohort and EST2, EIF251, SSRP1 and PARP1, in the second cohort.

## Discussion

Sixty differentially expressed TF and TR were retrieved from the microarray data published by Desai *et al.* (Desai *et al.*, 2004). These genes were assorted into 17 pathways by the DAVID knowledge base and into eight functional networks by Ingenuity Pathway Analysis (IPA). Though most these pathways were too general, and led to central metabolic hubs, such as PTEN (which was further validated in the parent study) and nuclear receptors pathways, some revealed particularly interesting and unforeseen aspects of prostate biology, such as circadian rhythms and peroxisome biogenesis. The selected genes were also mapped to chromosome regions frequently affected in prostate cancers, as their identification might serve as risk factors or therapeutic targets relevant to progression to CRPC. Additionally, 20 genes were found mutated in at least 5% of prostate adenocarcinoma patients.

We used the DAVID module Gene ID Conversion Tool (Huang *et al.*, 2007a, 2007b) to identify gene IDs from the initial gene list. The number of genes was roughly the same as those uncovered in the parent study, perhaps indicating a potential limit for retrieving information from the early commercially available microarray chips. In order to further advance our understanding of the physiology and endocrinology of the VP gland and to facilitate the biological interpretation of prostate biology in a broad range of biological processes, the 468 genes were further processed to track down their functional classification and resulted in 60 different TF or TR, which were then assigned to known regulatory pathways using DAVID and to functional interaction networks using IPA, resulting in the identification of 22 transcription factors.

Prostate cancer will affect one eighth to one sixth of men worldwide. In spite of enormous progress in the understanding of many facets of the disease, new concepts are still emerging. One of these is the clonal origin of metastases (Haffner *et al.*, 2013), punctuated rather than gradual progression (Baca *et al.*, 2013) and the non-linear relationship of on-site and distant metastases, meaning that metastases might be generated from less advanced local tumor foci (Haffner *et al.*, 2013). Accordingly, it is not completely understood as to which foci in the advanced stage of the disease will progress to CRPC. We followed the idea that the physiological adaptation of the gland to the hypoandrogenic castration-induced environment involves regulatory pathways to maintain the gland in a regressed, low proliferative and less functional (meaning less differentiated) state, and that these pathways in cancer cells might be corrupted and thereby contribute to the progression to CRPC. In this scenario, molecules with enhanced expression after castration might be found to be “tumor suppressors”, particularly if they function as hubs in regulatory networks that are defective in cancer cells. *Smad4*, *Ikbkb*, *Rara*, *ets1*, *Bhlhe41*, *Id2*, *Tnfrsf1a*, *Mxi1* and *Dbp* are new candidates, together with the well-known tumor suppressor *Pten*. As a matter of fact, these genes (except ID2 and Mxi1) were found deleted in prostate adenocarcinomas and advanced metastatic prostate cancers.

This also raises the question of whether *Pten*-defective tumors should be submitted to androgen deprivation or blockade, as a major aspect of the prostate response to falling androgen levels relies on this phosphatase.

The functional characterization of the 60 genes revealed interesting attributes. DAVID retrieved 17 pathways, most of them centered on either *Mapk1* or *Mapk9*, which is perhaps too general to indicate new physiological functions. It is worth noticing that MAPK pathway has been implicated in increased survival of castrate-resistant prostate cancer patients (Mukherjee *et al.*, 2011).

Next, we uncovered circadian rhythms as a relevant pathway, centered on the genes *Arntl* and *Bhlhe41*. These genes were included in network number 2 retrieved by IPA, which also included the *Dbp* gene, also implicated in circadian regulation. *Arntl* is directly linked to *Dbp* and indirectly to *Ar* via the p300 and CREBBP acetyl transferases. *Dbp* has been reported to have peak expression at 8 h within the light portion of the 12h:12h light/dark cycle (Zeitegeber, ZT 8) in the rat prostate gland (Qi *et al.*, 2009, Sunkel and Wang, 2014) in a similar fashion to other core clock genes in the mouse prostate (Bebas *et al.*, 2009). Moreover, *ARNTL* polymorphisms have been significantly associated with susceptibility to prostate cancer (Zhu *et al.*, 2009)^.^ This evidence raises the possibility that AR-dependent and AR-independent circadian functions contribute to the prostate gland physiology, by opening a new connection to environmental factors, knowingly significant in prostate cancer risk and incidence.

The peroxisome biogenesis pathway, represented by the sole gene Pex14, is also another connection to the environment, as peroxisome proliferation and activity are related to several environmental (and dietary) factors, adding further complexity to the peculiar metabolic adaptations of the gland given its function in accumulating citrate in secretions (Singh *et al.*, 2006).

We also found that some of the genes identified in the present investigation map to regions commonly deleted in prostate cancer. In particular, we refer to the *Pten/Hhex/Mxi1* cluster at 10q23, which was characterized in detail before (Hermans *et al.*, 2004). It will be interesting to investigate whether the differently sized deletions in this region might affect the behavior of prostate cancer cells, as *MXI1* is usually lacking or inactive in prostate cancer (Eagle *et al.*, 1995, Prochownik *et al.*, 1998); its function is to suppress proliferation by antagonizing *Myc* (Taj *et al.*, 2001), which in turn is commonly amplified in prostate cancer (Ishkanian *et al.*, 2009). It is important to mention that *Mxi1* expression is suggested to decay after castration, according to the parent study (Desai *et al.*, 2004). In contrast, *Hhex* expression is increased, which functions as a coordinator of hematopoiesis and the development of endoderm-derived organs such as the liver and thyroid (Martinez Barbera *et al.*, 2000).

Additionaly, we found the *Mapk1* gene, whose homolog *MAPK1* maps to 22q11.21, in a region between the 22q11.21 and 22q12.1 segments deleted in 29% and 33% of intermediate risk tumors, respectively, but not frequently observed in high risk cancers (Ishkanian *et al.*, 2009). The *MAPK1* gene product is better known as ERK-2 (or p42 MAPK) and funnels down a variety of extracellular signals to control several functions, particularly the G1-S transition within the cell cycle (Meloche and Pouysségur, 2007). The importance of MAPK1 is highlighted by the fact that it was enlisted as a node in 14 of the 17 pathways identified by DAVID, including prostate cancer among others, and the recent demonstration of the existence of identified mutations in members of the MAPK signaling pathway in the serum of 96% tested individuals harboring different tumors (Bettegowda *et al.*, 2014). Given the particular association of deletions in this region and the intermediate but not high risk of cancer, MAPK1 might be a protooncogene contributing to PCa progression, metastasis and/or transition to CRPC, and its deletion might represent a lower risk of disease progression.

Finally, we found 20 genes mutated in at least 5% of patients. In contrast to PTEN, mutations in these genes are secondary. However, it has been noted that prostate cancer is commonly associated with diverse low frequency mutations (Armenia *et al.*, 2018). Nonetheless, six of the identified genes (PHOX2a, NFYC, EST2, EIF251, SSRP1 and PARP1) were found associated with significant impact on patient survival.

The present analysis cannot distinguish between epithelial and stromal contributions to gene expression. Therefore, it is possible that some of the genes studied are expressed in the stroma. As a matter of fact, a previous approach from our laboratory has identified stromal and epithelial subsets of transcription factors (Nishan *et al.*, 2019).

In conclusion, this work provides insights into the vastness of physiological pathways involving multiple regulatory interactions among genes needed to adjust prostate biology to the reduced androgen levels achieved by surgical or chemical castration. These results are expected to help us understand the idiosyncrasies of prostate cancer.

## Supplementary material

The following online material is available for this article:

Table S1  - Functional associateions among the selected genes
