# Supplementary material for: Transcriptional regulators and regulatory pathways involved in prostate gland adaptation to a hypoandrogen environment
Source: Genet Mol Biol. 2020 Feb 14;42(4):e20180362. doi: 10.1590/1678-4685-GMB-2018-0362 (PMC7198032; doi:10.1590/1678-4685-GMB-2018-0362)
Supplement: Supplementary file 1 [file 1415-4757-GMB-42-4-e20180362-suppl.pdf]

Supplementary Material to "Transcriptional Regulators and Regulatory Pathways Involved in Prostate Gland Adaptation to a Hypoandrogen Environment"

Table S1 - Functional associations among the selected genes appearing in Figure 1.

ID	Molecules in Network	Score	Focus Molecules	Top Diseases and Functions
1	ANP32A, ATF2, CDK7, ERK1/2, ESR2, estrogen receptor, ETS1, GTF2B, Histone h3, HMGA2, Holo RNA polymerase II, ID2, IRF1, MAPK1, MAPK9, Mapk, N-cor, NFkB (complex), PARP1, PSMC5, PTEN, Rar, RARA, Rxr, RXRG, SMAD4, Smad2/3, SSRP1, TCEB1, THRB, thyroid hormone receptor, TXN, TXNIP, UBE2I, VDR	49	24	Gene Expression, Cell Death and Survival, Tumor Morphology<br>Nuclear receptor activity, centered in ESR2, RAR, THR and VDR
2	AR, ARNTL, Basal transcriptional machinery, BCKDHA, BHLHE41, BTG2, CALCA, CEBPB, Cebpd, CEBPG, CITED2, CREBBP, DBP, EP300, HIPK3, HOXB9, HOXD4, NFYC, NPAS2, NPR1, p300-CBP, PCMT1, PELP1, PHOX2A, RB1, SCT, SLC7A1, SNURF, Sox9, SP1, SRCAP, TAT, TIF2-NCOA1-p300-PCAF-CBP, TMPO, Vegf	15	10	Gene Expression, Cancer, Respiratory Disease<br>Connects circadian rhythms via Arntl and Dbp to androgen regulation via AR
3	Abcb1b, ACTR3, AGRN, CRIP2, DUSP4, DYNC1LI1, EGR1, EIF2AK1, EIF2S1, HAND1, HSPA2, HSPA8, HSPA9, Hspa1l, JAK3, JUN, KCNH2, KCNN1, LAMP2, LOC100360467/Mxi1, LTBP1, MAP1LC3B, MAPK3, MYOD1, NAB1, PKM, PTHLH, RALGAPA1, RPS14, RTCB, SERP1, SNCA, SOCS3, TGFB1, TUBB	13	9	Cell Death and Survival, Liver Necrosis/Cell Death, Cellular Development
4	ACTG1, Actin, Actin-Nrf2, ADRB2, ADRB3, AKT1, AKT3, CASP3, CD81, CRIP2, Dmd, DSTN, EHD4, FKBP5, FLNC, GCLC, GCLM, hexokinase, HHEX, HINT1, IKBKB, JUN, LOC290704, MAFG, MAFK, NFE2L2, PKN2, PTGFRN, RAB10, SH3GLB1, SRC, TNFRSF1A, TNS1, Ube2n, ZFP36L1	13	9	Cell Death and Survival, Inflammatory Disease, Respiratory Disease
5	A1CF, APOBEC1, cytidine deaminase	2	1	RNA Post-Transcriptional modification, Nucleic Acid Metabolism, Small Molecule Biochemistry<br>RNA C-to-U editing

ID	Molecules in Network	Score	Focus Molecules	Top Diseases and Functions
				function of APOBEC1
6	CRTC1, SIK1, STK11, STRADA	2	1	Carbohydrate Metabolism, Hereditary Disorder, Neurological Disease<br>Kinase activity of Sik1
7	HFE2, MYO10, NEO1, RGMA, TMPRSS6	2	1	Cell Morphology, Cellular Assembly and Organization, Hematological System Development and Function<br>Cell-cell, cell-matrix adhesion (via Neogenin 1) and proteolysis (via TMPRSS6 serine protease)
8	PEX5, PEX7, PEX13, PEX14, PEX19	2	1	Developmental Disorder, Hereditary Disorder, Metabolic Disease<br>Peroxisome biogenesis and function
